# Supplementary material for: A noncanonical AR addiction drives enzalutamide resistance in prostate cancer
Source: Nat Commun. 2021 Mar 9;12:1521. doi: 10.1038/s41467-021-21860-7 (PMC7943793; doi:10.1038/s41467-021-21860-7)
Supplement: Supplementary file 1 — Supplementary Information [file 41467_2021_21860_MOESM1_ESM.pdf]

Supplementary Information

A noncanonical AR addiction drives enzalutamide resistance in prostate cancer

Yundong He, Ting Wei, Zhenqing Ye, Jacob J. Orme, Dong Lin, Haoyue Sheng, Ladan Fazli, R. Jeffrey Karnes, Rafael Jimenez, Liguo Wang, Liewei Wang, Martin E. Gleave, Yuzhuo Wang, Lei Shi and Haojie Huang

Supplementary Fig.1

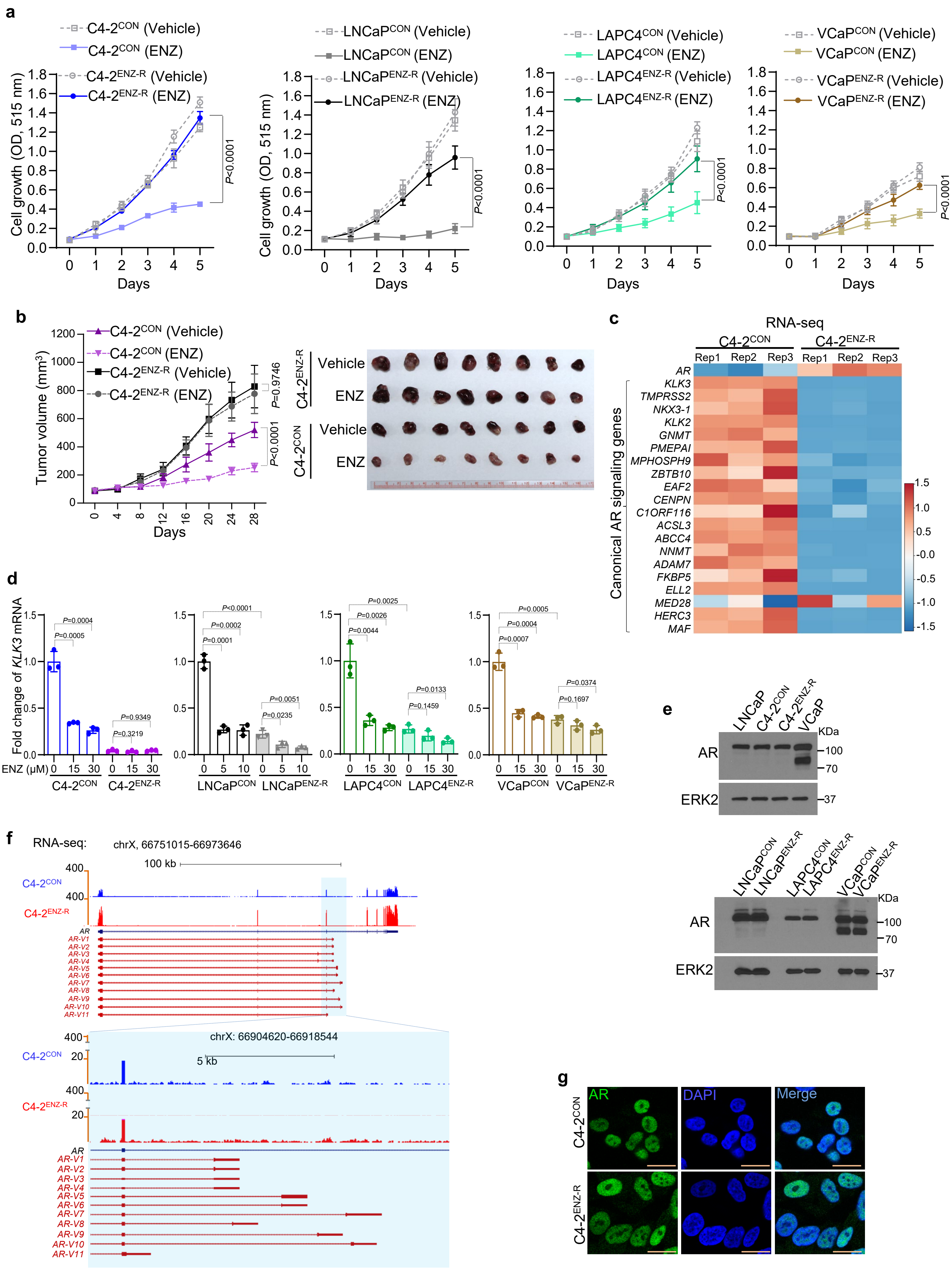

Supplementary Fig.1. Full-length AR but not AR variants is required for proliferation of ENZ-resistant cells. a C4-2^{CON}, C4-2^{ENZ-R}, $LNCaP^{ENZ-R}$, $LAPC4^{CON}$, $LAPC4^{ENZ-R}$, $VCaP^{CON}$ and $VCaP^{ENZ-R}$ cells were treated with 10 μM ($LNCaP^{CON}$ and $LNCaP^{ENZ-R}$), 30 μM (C4-2^{CON}, C4-2^{ENZ-R} , $LAPC4^{CON}$, $LAPC4^{ENZ-R}$, $VCaP^{CON}$ and $VCaP^{ENZ-R}$) ENZ or vehicle for different periods of time and cell proliferation was measured using SRB assay. Data are represented as means ± s.d., (n = 6 replicates/group). Statistical significance was determined by two-way ANOVA. **b** Growth of C4-2^{CON} and C4-2^{ENZ-R} xenografts was assessed over 28 days in mice treated with vehicle or ENZ. Means ± s.d., (n = 8 replicates/group). Statistical significance was determined by two-way ANOVA. **c** Heatmap showing RNA-seq read intensity (expression) of the canonical AR signature genes in C4-2^{CON} and C4-2^{ENZ-R} cells. **d** Real time RT-PCR showing the mRNA level of *KLK3* in C4-2^{CON}, C4-2^{ENZ-R}, $LNCaP^{CON}$, $LNCaP^{ENZ-R}$, $LAPC4^{CON}$, $LAPC4^{ENZ-R}$, $VCaP^{CON}$ and $VCaP^{ENZ-R}$ cells treated with the indicated concentrations of ENZ for 24 h. Data are represented as means ± s.d., (n = 3 replicates/group). Statistical significance was determined by unpaired two-tailed Student's t test. **e** Western blot showing the level of full-length (FL) AR and AR variants (Vs) in LNCaP, C4-2^{CON}, C4-2^{ENZ-R} and VCaP cell lines (top); Western blot showing the level of full-length (FL) AR and AR variants (Vs) in $LNCaP^{CON}$, $LNCaP^{ENZ-R}$, $LAPC4^{CON}$, $LAPC4^{ENZ-R}$, $VCaP^{CON}$ and $VCaP^{ENZ-R}$ cell lines (bottom); ERK2 was used as a loading control; Experiments were repeated twice. **f** UCSC tracks (integration of three replicates) showing RNA-seq signals of WT *AR* gene and *AR* variants in C4-2^{CON} and C4-2^{ENZ-R} cells. RNA-seq data show that no obvious expression of *AR* variants was detected in C4-2^{CON} and C4-2^{ENZ-R} cells. **g** Immunofluorescence (IF) of AR and DAPI in C4-2^{CON} and C4-2^{ENZ-R} cells with anti-AR C-terminal antibody (C-19). Scale bar, 10 μm; Experiments were repeated twice.

Supplementary Fig.2

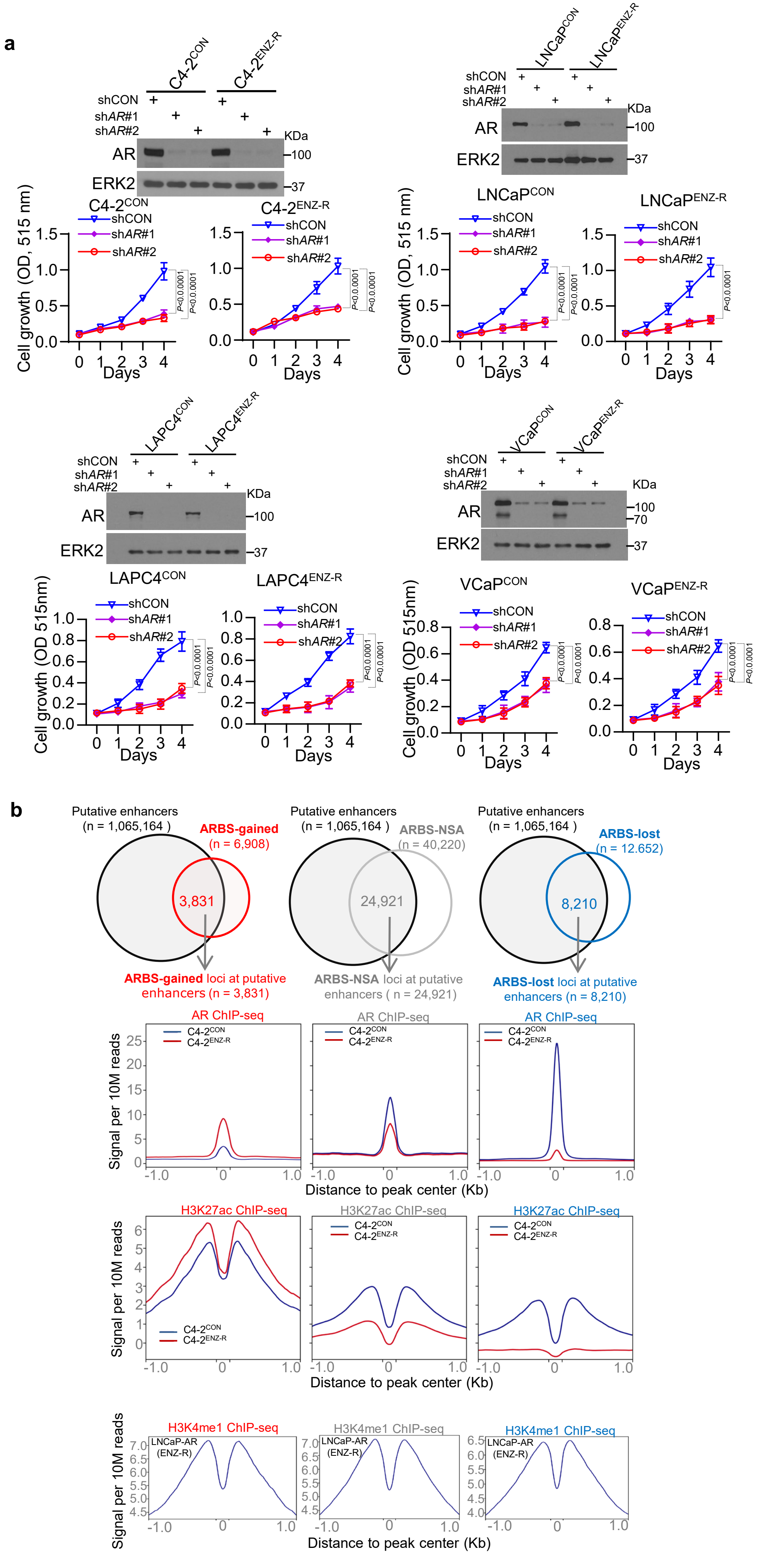

Supplementary Fig.2. AR is required for proliferation of ENZ-resistant cells. a *AR* was knocked down by specific shRNAs in C4-2^{CON}, C4-2^{ENZ-R}, LNCaP^{CON}, LNCaP^{ENZ-R}, LAPC4^{CON}, LAPC4^{ENZ-R}, VCaP^{CON} and VCaP^{ENZ-R} cells and cell proliferation was measured using SRB assay. Data are represented as means ± s.d., (n = 5 replicates/group). Statistical significance was determined by two-way ANOVA. **b** Analysis of AR binding signal and H3K27ac enrichment at putative enhancers in C4-2^{CON} and C4-2^{ENZ-R} cells. ChIP-seq data of H3K4me1 histone mark (publicly available data, Gene Expression Omnibus GSE103449) in ENZ-resistant LNCaP-AR cells was used for the reference of putative enhancers.

Supplementary Fig.3

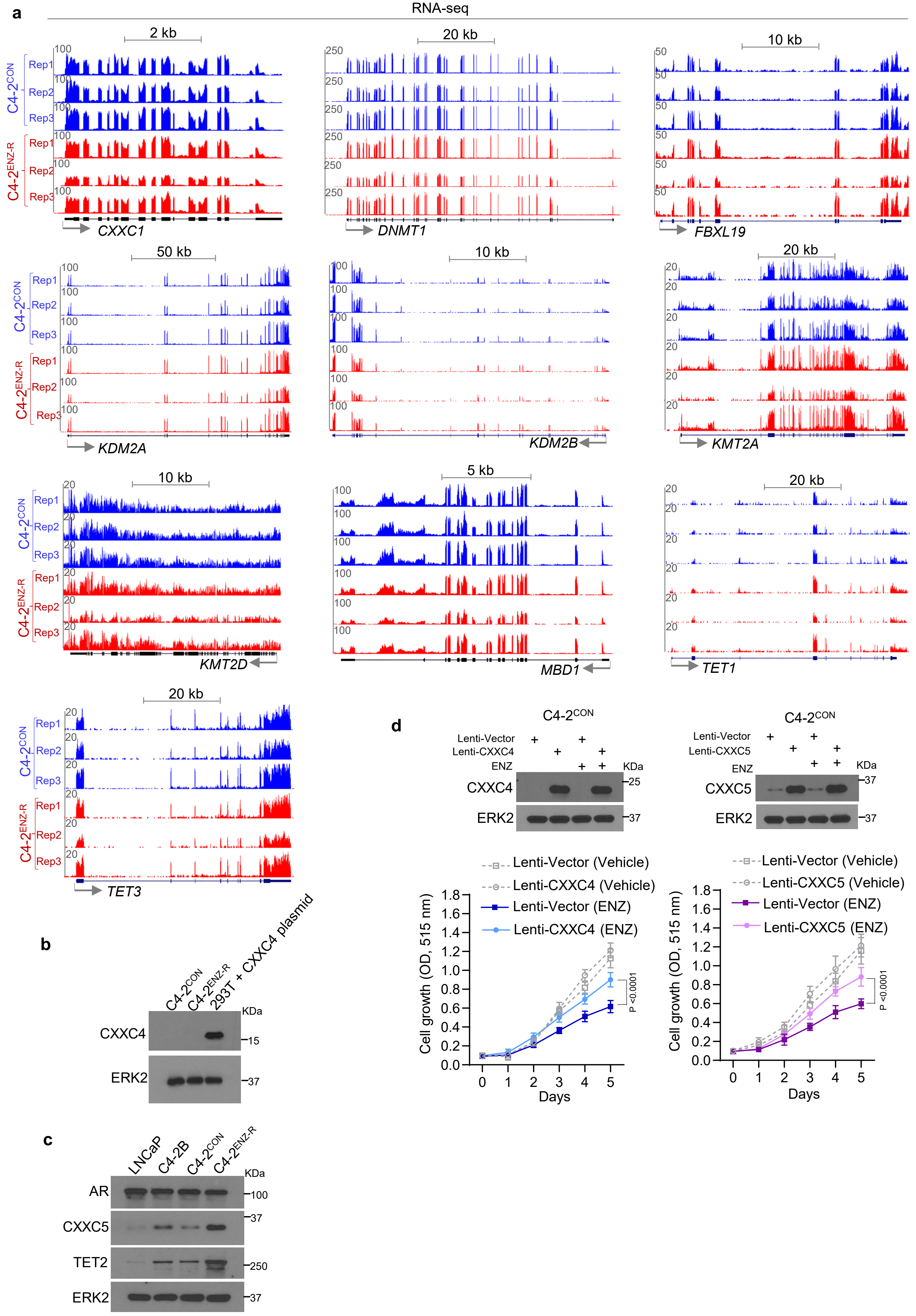

Supplementary Fig.3. RNA-seq data shows expression of CXXC domain genes in C4-2^{CON} and C4-2$^{ENZ\text{-}R}$ cells. a UCSC tracks showing RNA-seq signals of *KDM2A*, *KDM2B*, *FBXL19*, *CXXC1*, *DNMT1*, *KMT2A*, *KMT2D, MBD1*, *TET1* and *TET3* genes in C4-2^{CON} and C4-2$^{ENZ\text{-}R}$ cells. **b** Western blot showing the expression of CXXC4 protein in C4-2^{CON} and C4-2$^{ENZ\text{-}R}$. 293T cells transfected with CXXC4 plasmid was used as positive control. ERK2 was used as a loading control; Experiments were repeated twice. **c** Western blotting showing the AR, CXXC5, and TET2 protein level in the indicated cell lines. Experiments were repeated twice. **d** C4-2^{CON} cells were transfected with lentiviral expression vector for CXXC4 or CXXC5 and treated with 10 μM ENZ or vehicle (DMSO) for different periods of time and cell proliferation was measured using SRB assay. Data are represented as means ± s.d., (n = 6 replicates/group). Statistical significance was determined by two-way ANOVA.

Supplementary Fig.4

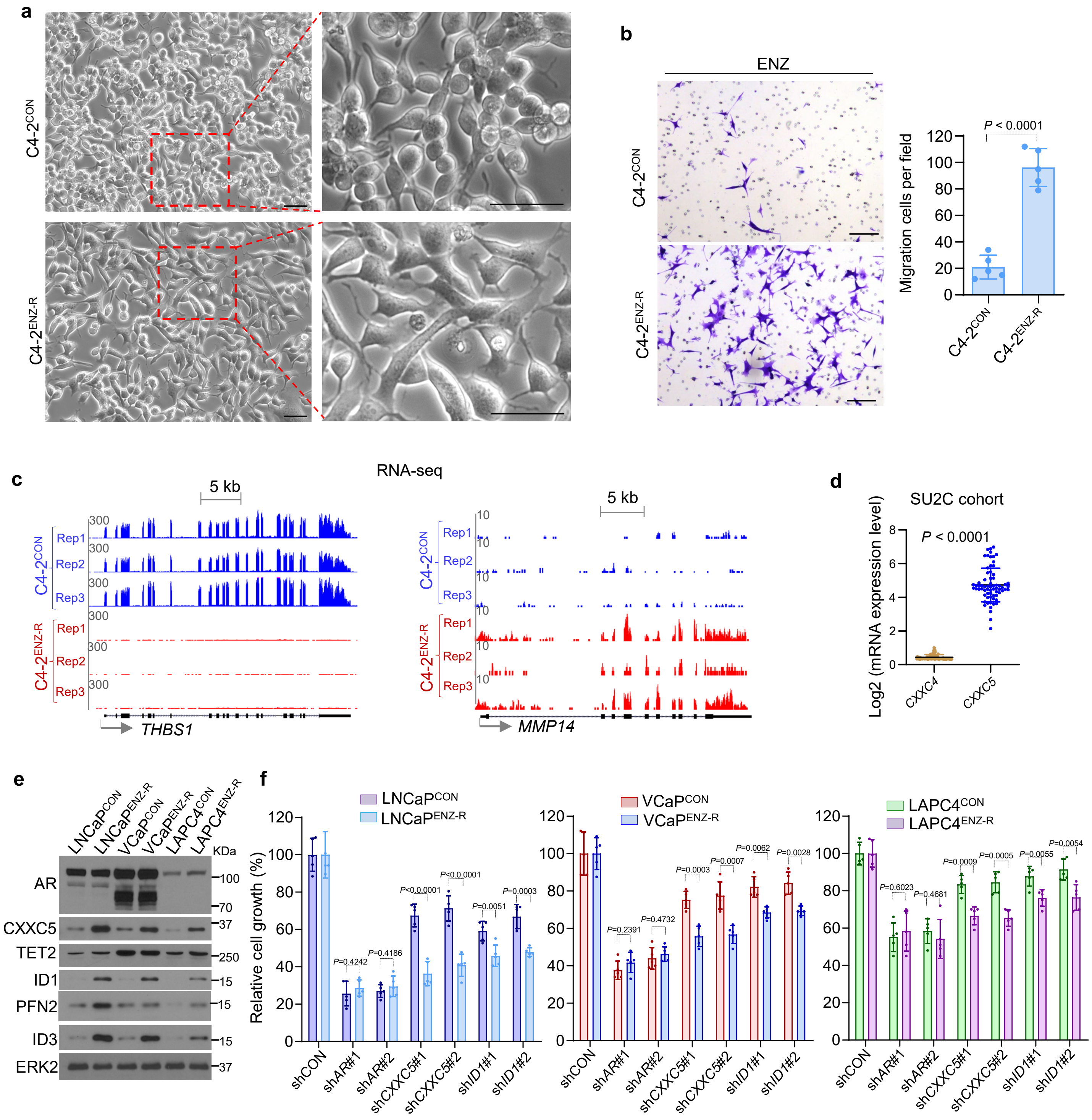

Supplementary Fig.4. Role of noncanonical AR target genes in ENZ resistance in CRPC cells. a C4-2^{CON} and C4-2^{ENZ-R} cells grew at logarithmic phase were photographed. Scale bar, 50 µm. **b** C4-2^{CON} and C4-2^{ENZ-R} cells were treated with 30 µM of ENZ in Transwell chambers for 24 h and the migrated cells were photographed and counted. Scale bar, 50 µm. Data are represented as means ± s.d., (n = 3 replicates/group). Statistical significance was performed by unpaired two-tailed Student's t test. **c** UCSC tracks showing RNA-seq signals of *THBS1* and *MMP14* genes in C4-2^{CON} and C4-2^{ENZ-R} cells. **d** Comparison of *CXXC4* and *CXXC5* mRNA level in CRPC treated with AR signaling inhibitors (including ENZ and ABI) from the SU2C cohort. **e** Western blot analysis of AR, CXXC5, TET2, ID1, PFN2 and ID3 proteins in control and ENZ-R LNCaP, VCaP or LAPC4 cells. ERK2 was used as a loading control; Experiments were repeated twice. **f** Relative cell proliferation of control (CON) and ENZ-R LNCaP, VCaP or LAPC4 cells infected with the indicated lentivirus expressing the indicated shRNAs. Means ± s.d., (n = 5 replicates/group). Statistical significance was determined by unpaired two-tailed Student's t tests.

Supplementary Fig.5

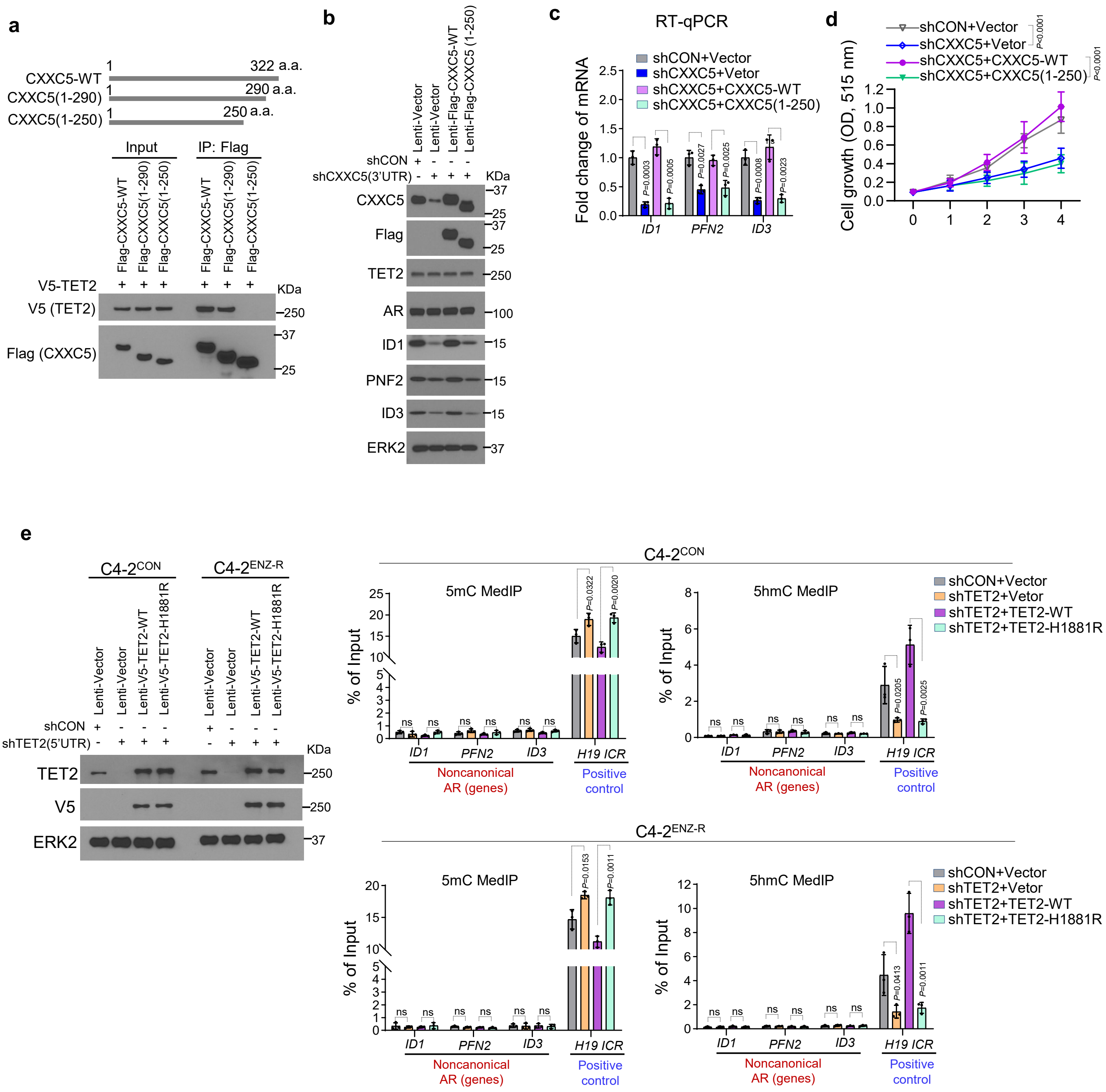

Supplementary Fig.5. Effect of TET2 on DNA methylation at the noncanonical AR target gene loci. a Co-IP analysis of TET2 interaction with WT CXXC5 or C-terminal truncation mutants in 293T cells. Experiments were repeated twice. **b** Western blot showing the expression level of indicated proteins in C4-2^{ENZ-R} cells with knockdown of endogenous CXXC5 and restored expression of CXXC5-WT or CXXC5(1-250). ERK2 was used as a loading control; Experiments were repeated twice. **c** RT-qPCR analysis of expression of *ID1, PFN2* and *ID3* genes in C4-2^{ENZ-R} cells with knockdown of endogenous CXXC5 and restored expression of CXXC5-WT or CXXC5(1-250). Data shown as means ± s.d. (n = 3 replicates/group). Statistical significance was determined by unpaired two-tailed Student's t tests. **d** Cell proliferation analysis of C4-2^{ENZ-R} cells with knockdown of endogenous CXXC5 and restored expression of CXXC5-WT or CXXC5(1-250). Data shown as means ± s.d. (n = 6 replicates/group). Statistical significance was determined by two-way ANOVA. **e** MedIP assay to detect the 5mC/5hmC level at the noncanonical AR target gene loci *ID1, PFN2* and *ID3* in C4-2^{CON}/C4-2^{ENZ-R} cells with knockdown of endogenous TET2 and restored expression of TET2-WT or TET2-H1881R. The *H19* imprinting control region (ICR) was used as a positive control. Data shown as means ± s.d. (n = 3 replicates/group). Statistical significance was determined by unpaired two-tailed Student's t tests.

Supplementary Fig.6

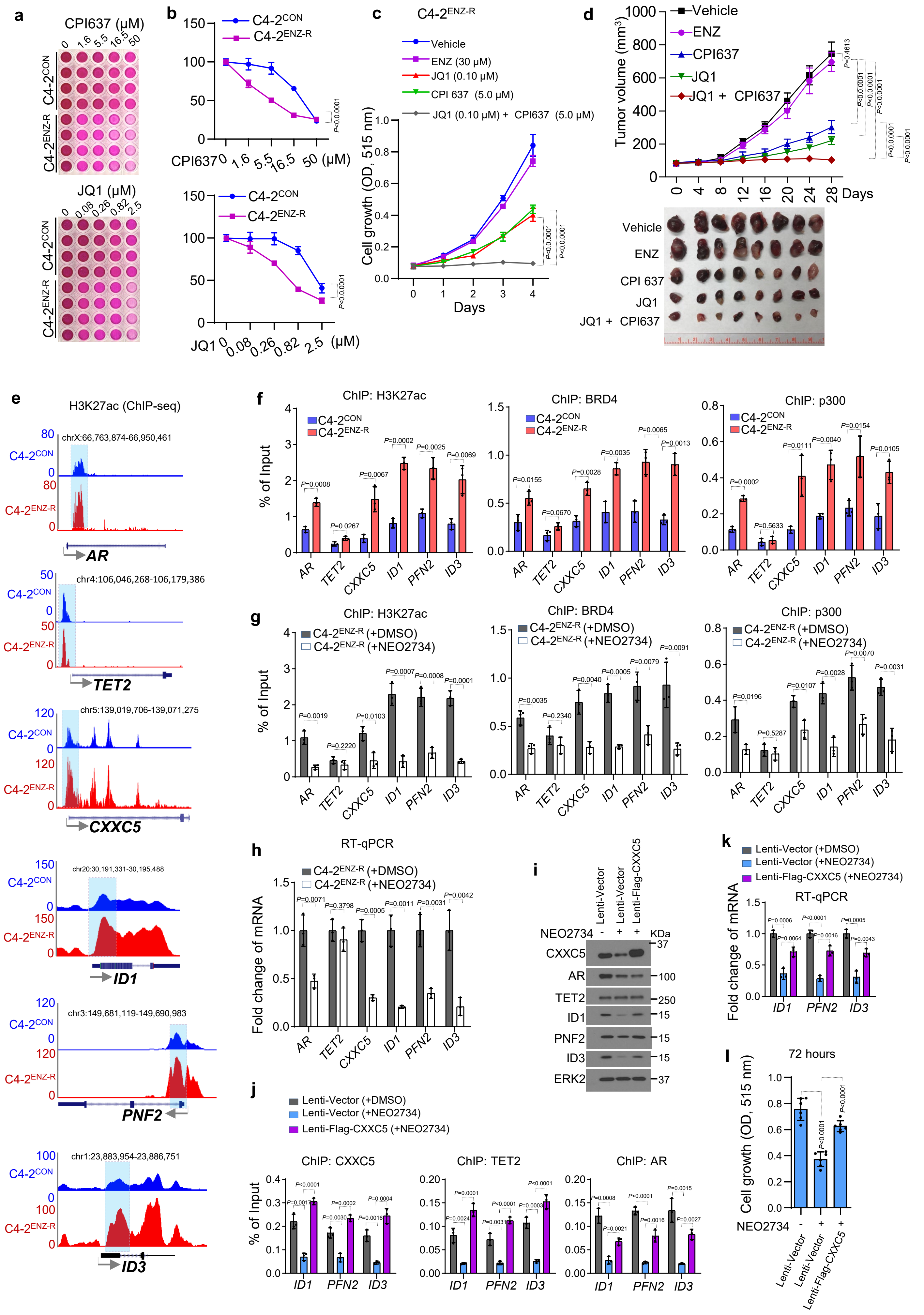

Supplementary Fig.6. Sensitivity of ENZ-resistant CRPC to BET or CBP/p300 inhibitor or their combination. a, b C4-2^{CON} and C4-$2^{ENZ\text{-}R}$ cells were treated with the indicated concentrations of CBP/p300 inhibitor (CPI637) or BET inhibitor (JQ1) for 4 days and cell proliferation was measured using SRB assay and the 96-well plates were photographed. Data are represented as means ± s.d., (n = 4 replicates/group). Statistical significance was determined by two-way ANOVA. **c** C4-$2^{ENZ\text{-}R}$ cells were treated with the indicated concentrations of ENZ, BET inhibitor (JQ1), CBP/p300 inhibitor (CPI637) or combination of JQ1 and CPI-637 for the indicated periods of time and cell proliferation was measured using SRB assay. Data are represented as means ± s.d., (n = 6 replicates/group). Statistical significance was determined by two-way ANOVA. **d** C4-$2^{ENZ\text{-}R}$ cells suspended in 0.1 ml Matrigel were injected into the right flank of 6-week-old male SCID mice. After the volume of tumors reached about 100 mm^3, mice were randomly assigned to the indicated groups and mice were orally administrated with 10 mg/kg ENZ, 10 mg/kg CPI637, 50 mg/kg JQ1, combination of 50 mg/kg JQ1 and 10 mg/kg CPI637 or vehicle per day and tumor size was measured every 4 days (**top**). After 28 days of treatment, tumors from the indicated groups were photographed (**bottom**). Data are represented as means ± s.d., (n = 8 replicates/group). Statistical significance was determined by two-way ANOVA. **e** UCSC tracks (integration of three replicates) showing ChIP-seq signals of H3K27ac at genomic loci of *AR, TET2, CXXC5, ID1, PFN2* and *ID3* genes in C4-2^{CON} and C4-$2^{ENZ\text{-}R}$ cells. **f** ChIP-qPCR analysis of H3K27ac, BRD4 and p300 occupancy at genomic loci of *AR, TET2, CXXC5, ID1, PFN2* and *ID3* genes in C4-2^{CON} and C4-$2^{ENZ\text{-}R}$ cells. Data shown as means ± s.d. (n = 3 replicates/group). Statistical significance was determined by unpaired two-tailed Student's t tests. **g** ChIP-qPCR analysis of H3K27ac, BRD4 and p300 occupancy at genomic loci of *AR, TET2, CXXC5, ID1, PFN2* and *ID3* genes in C4-$2^{ENZ\text{-}R}$ cells treated with DMSO or 50 nM NEO2734. Data shown as means ± s.d. (n = 3 replicates/group). Statistical significance was determined by unpaired two-tailed Student's t tests. **h** RT-qPCR analysis of expression of *AR, TET2, CXXC5, ID1, PFN2* and *ID3* genes in C4-$2^{ENZ\text{-}R}$ cells treated with DMSO or 50 nM NEO2734. Data shown as means ± s.d. (n = 3 replicates/group). Statistical significance was determined by unpaired two-tailed Student's t tests. **i** Western blot showing the expression level of indicated proteins in C4-$2^{ENZ\text{-}R}$ cells with or without overexpression of CXXC5 and treatment of DMSO or 25 nM NEO2734 for 24 hours. Experiments were repeated twice. **j** ChIP-qPCR analysis of CXXC5, TET2 and AR occupancy at genomic loci of *ID1, PFN2* and *ID3* genes in C4-$2^{ENZ\text{-}R}$ cells with or without overexpression of CXXC5 and treatment of DMSO or 25 nM NEO2734 for 24 hours. Data shown as means ± s.d. (n = 3 replicates/group). Statistical significance was determined by unpaired two-tailed Student's t tests. **k** RT-qPCR analysis of expression of *ID1, PFN2 and ID3* in C4-$2^{ENZ\text{-}R}$ cells with or without overexpression of CXXC5 and treatment of DMSO or 25 nM NEO2734 for 24 hours. Data shown as means ± s.d. (n = 3 replicates/group). Statistical significance was determined by unpaired two-tailed Student's t tests. **l** Cell proliferation analysis of C4-$2^{ENZ\text{-}R}$ cells with or without overexpression of CXXC5 and treatment of DMSO or 25 nM NEO2734 for 72 hours. Data shown as means ± s.d. (n = 6 replicates/group). Statistical significance was determined by unpaired two-tailed Student's t tests.

Supplemental Fig.7

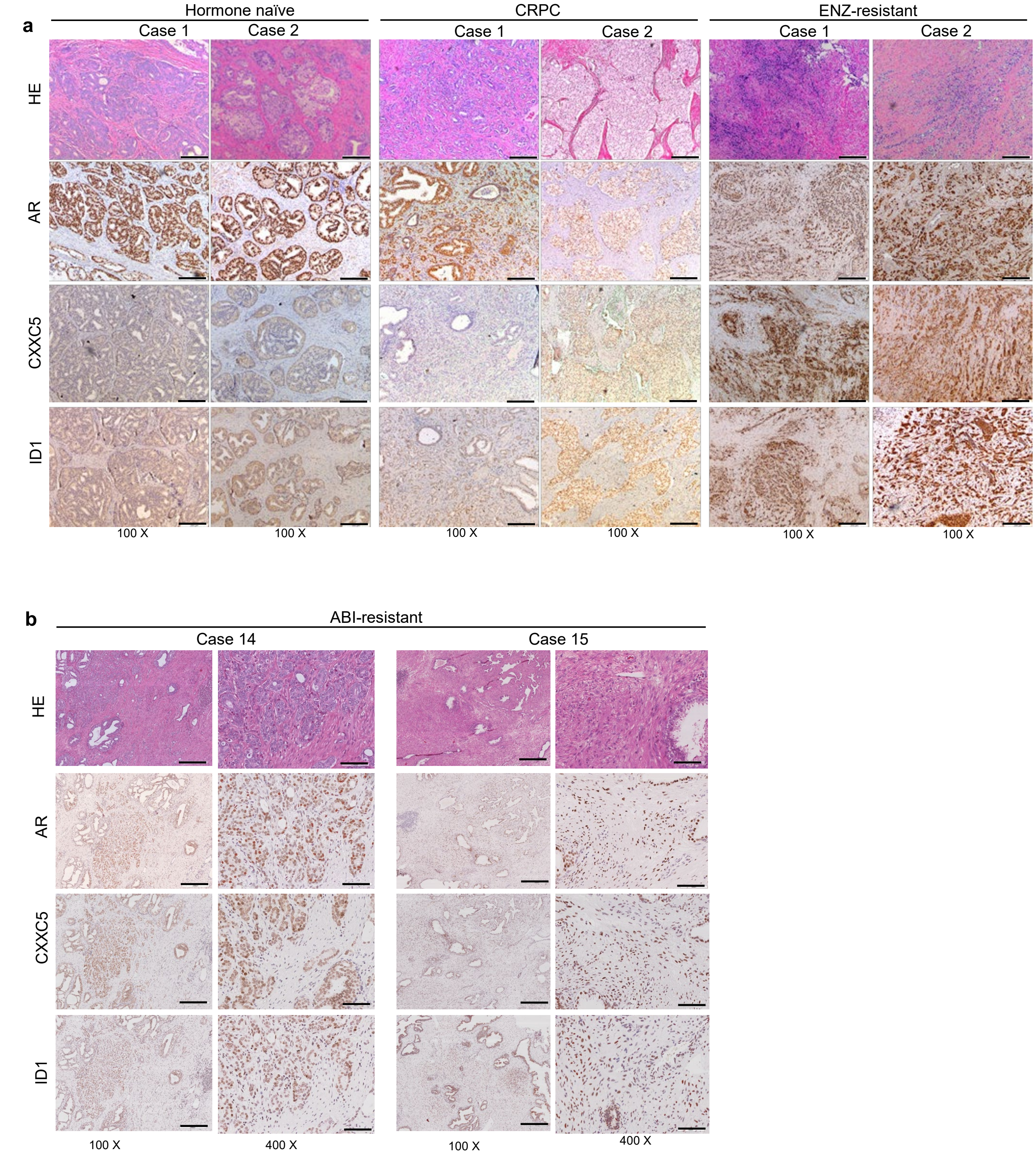

Supplementary Fig.7. Upregulation of CXXC5 and ID1 in CRPC patients treated with AR pathway inhibitors. a HE staining and IHC of AR, CXXC5 and ID1 proteins in tumor tissue of hormone naïve (n = 24), CRPC (n = 16) and ENZ-resistant (n = 13) PCa patients; representative images at low magnification (scale bar, 200 µm) are shown. **b** HE staining and IHC of AR, CXXC5 and ID1 proteins in tumor tissues of ABI-resistant PCa patients; representative images [scale bar, 200 µm (100 ×); 50 µm (400 ×)] are shown. Note: See the IHC staining sore in Supplementary Data 2; IHC was scored by two investigators independently.

Supplemental Fig.8

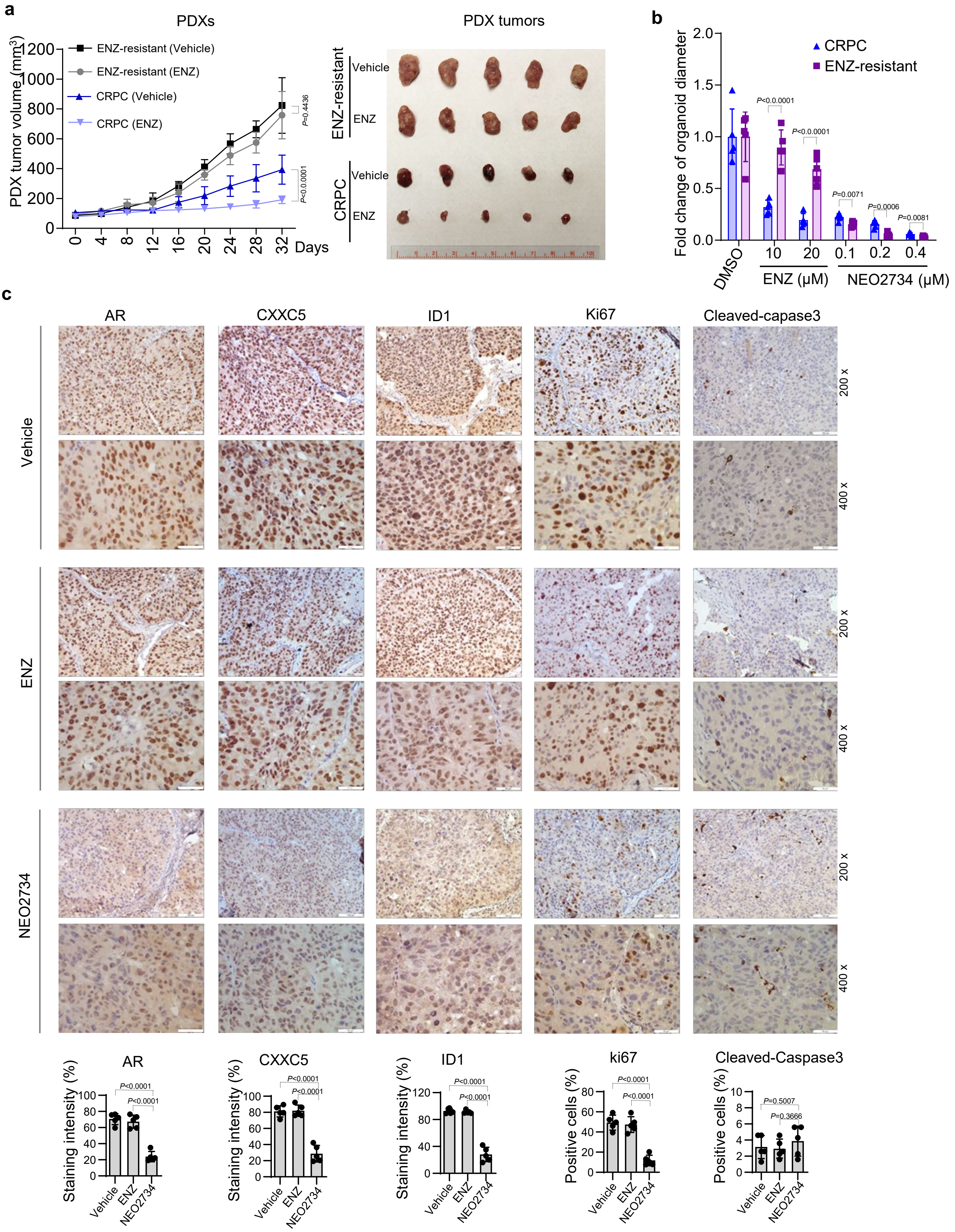

Supplementary Fig.8. Inhibition of noncanonical AR activity in ENZ-resistant PDX by BET-CBP/p300 dual inhibitor NEO2734. a Effect of ENZ treatment on the growth of CRPC PDXs and ENZ-resistant PDXs. CRPC and ENZ-resistant PDXs were planted into the right flank of 6-week-old male SCID mice. After the volume of PDX tumors reached about 100 mm^3, mice were randomly assigned to the indicated groups and mice were orally administrated with 10 mg/kg ENZ per day or vehicle and tumor size was measured every 4 days (**left**). After 32 days of treatment, tumors from the indicated group were photographed (**right**). Data are represented as means ± s.d., (n = 5 replicates/group). Statistical significance was determined by two-way ANOVA. **b** Organoids from CRPC and ENZ-resistant PDXs were treated with the indicated inhibitors for 10 days and the diameter of organoids was measured. Data are represented as means ± s.d., (n = 5 replicates/group). Statistical significance was performed by unpaired two-tailed Student's t test. **c** IHC of AR, CXXC5, ID1, PFN2, Ki-67 and cleaved-caspase3 in ENZ-resistant PDXs treated for 36 days with vehicle, ENZ or NEO2734; representative images [scale bar, 100 µm (200 ×); 50 µm (400 ×)] were taken (**top**) and quantified data are shown in (**bottom**). Data are represented as means ± s.d., (n = 5 replicates/group). Statistical significance was performed by unpaired two-tailed Student's t test.

Supplementary Table 1. ARBS overlapped with CpGi

	Total_peaks	Promoter	None-promoter	Total_ peaks overlapped with CpGI	Peaks overlapped with CpGI at promoter	Peaks overlapped with CpGI at none-promoter
ARBS-gained	6908	841	6067	1293 (18.7%)	693 (82.4%)	600 (0.98%)
ARBS-NSA	40220	1371	38849	1276 (3.1%)	433 (31.8 %)	843 (0.22%)
ARBS-lost	12652	166	12486	15 (0.52%)	2 (1.2%)	13 (0.001%)
