# Supplementary material for: A noncanonical AR addiction drives enzalutamide resistance in prostate cancer
Source: Nat Commun. 2021 Mar 9;12:1521. doi: 10.1038/s41467-021-21860-7 (PMC7943793; doi:10.1038/s41467-021-21860-7)
Supplement: Supplementary file 3 — Description of Additional Supplementary Files [file 41467_2021_21860_MOESM3_ESM.pdf]

Description of Additional Supplementary Files

Title: Supplementary Data 1.

Description: Upregulated genes associated with ARBS-gained CpGi. Differential gene expression analysis was conducted using edgeR (version 3.6.8).

Title: Supplementary Data 2.

Description: IHC staining score of AR, CXXC5 and ID1 proteins in prostate cancer patient samples (see IHC scoring details in Methods section).

Title: Supplementary Data 3.

Description: Sequence information for primers, siRNAs and shRNAs.
