# Supplementary material for: A noncanonical AR addiction drives enzalutamide resistance in prostate cancer
Source: Nat Commun. 2021 Mar 9;12:1521. doi: 10.1038/s41467-021-21860-7 (PMC7943793; doi:10.1038/s41467-021-21860-7)
Supplement: Supplementary file 7 — Reporting Summary [file 41467_2021_21860_MOESM7_ESM.pdf]

nature research

Corresponding author(s): Haojie Huang

Last updated by author(s): 2021-2-6

Reporting Summary

Nature Research wishes to improve the reproducibility of the work that we publish. This form provides structure for consistency and transparency in reporting. For further information on Nature Research policies, see our Editorial Policies and the Editorial Policy Checklist.

Statistics

For all statistical analyses, confirm that the following items are present in the figure legend, table legend, main text, or Methods section.

n/a	Confirmed	
☐	☒	The exact sample size (*n*) for each experimental group/condition, given as a discrete number and unit of measurement
☐	☒	A statement on whether measurements were taken from distinct samples or whether the same sample was measured repeatedly
☐	☒	The statistical test(s) used AND whether they are one- or two-sided<br>*Only common tests should be described solely by name; describe more complex techniques in the Methods section.*
☒	☐	A description of all covariates tested
☒	☐	A description of any assumptions or corrections, such as tests of normality and adjustment for multiple comparisons
☐	☒	A full description of the statistical parameters including central tendency (e.g. means) or other basic estimates (e.g. regression coefficient) AND variation (e.g. standard deviation) or associated estimates of uncertainty (e.g. confidence intervals)
☐	☒	For null hypothesis testing, the test statistic (e.g. *F*, *t*, *r*) with confidence intervals, effect sizes, degrees of freedom and *P* value noted<br>*Give P values as exact values whenever suitable.*
☒	☐	For Bayesian analysis, information on the choice of priors and Markov chain Monte Carlo settings
☒	☐	For hierarchical and complex designs, identification of the appropriate level for tests and full reporting of outcomes
☒	☐	Estimates of effect sizes (e.g. Cohen's *d*, Pearson's *r*), indicating how they were calculated

Our web collection on statistics for biologists contains articles on many of the points above.

Software and code

Policy information about availability of computer code

Data collection	GraphPad Prism version 7.0 (https://graphpad-prism.software.informer.com/7.0/) was used for cell proliferation data, RT-PCR data and tumor volume data collection and analysis.<br>Image-Pro Plus 6.0 (https://image-pro-plus.software.informer.com/6.0/) was used for IHC data collection and analysis.

Data analysis	ChIP-seq data analysis:<br>1) bowtie2 v2.1.0 with default parameters was used for reads mapping to GRCh37 (hg19). (https://sourceforge.net/projects/bowtie-bio/files/bowtie2/2.1.0/)<br>2) callpeak function with "-p 1e-5" in MACS2 v2.0.10 was used for peaks calling. (https://pypi.org/project/MACS2/2.0.10.20131216/)<br>3) annotatePeaks.pl in HOMER v4.11 was used for peaks' genomic distribution annotation with regard to TSS and target genes. (http://homer.ucsd.edu/homer/ngs/annotation.html)<br>4) STEME v1.9.6 was used for de novo motif discovery. (https://pypi.org/project/STEME/#files)<br>5) Tools of computeMatrix and plotHeatmap in deepTools 2.0 were used for ChIP-seq intensity heatmap. (https://deeptools.readthedocs.io/en/develop/)<br><br>RNA-seq data analysis:<br>1) TopHat v2.0.9 with default options was used for reads alignment to GRCh37 (hg19). (https://tophat2-and-bowtie-compatibility.readthedocs.io/en/latest/)<br>2) FPKM_count.py in RSeQC v4.0.0 was used for gene expression counts. (http://rseqc.sourceforge.net/)<br>3) edgeR v3.6.8 was used for significant differential gene expression analysis. (https://bioconductor.statistik.tu-dortmund.de/packages/2.14/bioc/html/edgeR.html)<br>4) GSEA pre-ranked mode with default settings was used for gene expression enrichment analysis. (http://software.broadinstitute.org/gsea/msigdb/annotate.jsp)

For manuscripts utilizing custom algorithms or software that are central to the research but not yet described in published literature, software must be made available to editors and reviewers. We strongly encourage code deposition in a community repository (e.g. GitHub). See the Nature Research guidelines for submitting code & software for further information.

Data

Policy information about availability of data

All manuscripts must include a data availability statement. This statement should provide the following information, where applicable:

- Accession codes, unique identifiers, or web links for publicly available datasets
- A list of figures that have associated raw data
- A description of any restrictions on data availability

The RNA-seq and ChIP-seq data are deposited in the National Center for Biotechnology Information (NCBI) Gene Expression Omnibus (GEO) database with the accession code GSE136130: https://www.ncbi.nlm.nih.gov/geo/query/acc.cgi?acc=GSE136130.

Field-specific reporting

Please select the one below that is the best fit for your research. If you are not sure, read the appropriate sections before making your selection.

☒ Life sciences ☐ Behavioural & social sciences ☐ Ecological, evolutionary & environmental sciences

For a reference copy of the document with all sections, see nature.com/documents/nr-reporting-summary-flat.pdf

Life sciences study design

All studies must disclose on these points even when the disclosure is negative.

Sample size	An N of 5 to 10 mice was used for each condition in each experiment, based on standard practice in the field and prior experience working with the specified mouse models. No explicit power calculation was performed.
Data exclusions	Animals were excluded from analysis only if they became ill or their weight dropped below 90% of their original weight at the start of the experiment. However, no animals were excluded from the current study.
Replication	Replicates of experiments are specified in figure legends and two-tailed student T-test was used to determine if the differences were statistically significant unless specified otherwise.
Randomization	In mouse experiments, all mice were randomly stratified into groups, with littermates evenly distributed across conditions. Such concepts are irrelevant to the other in vitro and ex vivo experiments, because all known variables were controlled for in those settings.
Blinding	For mouse study, the tumor growth in mice was measured blindly and the investigators were blinded to the group allocation. For other studies, researchers were not blinded because it was not feasible to conduct truly blinded experiments with the laboratory staff available.

Reporting for specific materials, systems and methods

We require information from authors about some types of materials, experimental systems and methods used in many studies. Here, indicate whether each material, system or method listed is relevant to your study. If you are not sure if a list item applies to your research, read the appropriate section before selecting a response.

Materials & experimental systems

n/a	Involved in the study	
	x	Antibodies
	x	Eukaryotic cell lines
x		Palaeontology and archaeology
	x	Animals and other organisms
	x	Human research participants
x		Clinical data
	x	Dual use research of concern

Methods

n/a	Involved in the study	
	x	ChIP-seq
x		Flow cytometry
x		MRI-based neuroimaging

Antibodies

Antibodies used

For ChIP and ChIP-seq, antibodies were AR (2 µg/sample; #sc-816, Santa Cruz Biotechnology), FOXA1 (2 µg/sample; #ab23738, Abcam), H3K27ac (2 µg/sample; #ab4729, Abcam), CXXC5 (2 µg/sample; #16513-1-AP, Proteintech), TET2 (2 µg/sample; #ab94580, Abcam) and Rabbit IgG (2 µg/sample; #ab171870, Abcam).
For IHC analysis, primary antibodies were CXXC5 (dilution 1:500; #16513-1-AP, Proteintech), AR (dilution 1:1000; #ab108341, Abcam), ID1 (dilution 1:1000; #ab66495, Abcam) and PFN2 (1:1000; #LS-C186004-100, LSBio).
For western blotting or dot blotting, primary antibodies were AR (dilution 1:1000; #sc-816, Santa Cruz Biotechnology), CXXC5 (dilution 1:1000; #16513-1-AP, Proteintech), CXXC4 (dilution 1:500; #ab105400, Abcam), TET2 (dilution 1:1000; #MABE462, Millipore), TET3 (dilution 1:1000; #ab139311, Abcam), TET1 (dilution 1:1000; #ab191698, Abcam), ID3 (dilution 1:500, #sc-56712, Santa Cruz Biotechnology), PFN2 (dilution 1:1000; #sc-100955, Santa Cruz Biotechnology), BRD4 (dilution 1:1000; #ab128874, Abcam), p300 (dilution 1:1000; #MS-586-PO, Thermo Scientific), ID1 (dilution 1:1000; #ab66495, Abcam), FOXA1 (dilution 1:1000; #ab23738, Abcam), Flag (dilution 1:1000; #F1804, Sigma Aldrich) and V5 (dilution 1:1000; #A190-120A, Bethyl Laboratories) and ERK2 (dilution 1:2000; #sc-1647, Santa Cruz Biotechnology), biotinylated goat-anti-rabbit IgG secondary antibodies (dilution 1:1000; #BA-9200, Vector Labs), 5hmC antibody (dilution 1:1000; #39769, Active Motif), HRP-conjugated IgG secondary antibody (dilution: 1:10000; #7074, Cell Signaling Technology).
For MeDIP, primary antibodies were 5hmC (0.5 µg/sample, #39769, Active Motif), 5mC (1 µg/samples, #39649, Active Motif).

Validation

All of the antibodies used in this study were validated for the use in human specimens by the manufacturers and for the respective methods used in this manuscript (see home pages of respective manufacturers using catalogue numbers provided above).

Eukaryotic cell lines

Policy information about cell lines

Cell line source(s)

LNCaP, VCaP and LAPC4 prostate cancer cell lines and 293T cell line were purchased from The American Type Culture Collection (ATCC). C4-2 cells were purchased from Uro Corporation (Oklahoma City, OK).

Authentication

The cell lines were authenticated periodically via STR profiling (IDEXX BioResearch).

Mycoplasma contamination

Mycoplasma contamination was tested by the PCR Mycoplasma Detection Set (Takara, Otsu, Japan). All cell lines are negative for mycoplasma contamination.

Commonly misidentified lines
(See ICLAC register)

These cell lines were not used.

Animals and other organisms

Policy information about studies involving animals; ARRIVE guidelines recommended for reporting animal research

Laboratory animals

6-week old SCID male mice were used for xenograft study as described in the section of Methods. All mice were housed under standard conditions room temperature with a 12 h light/dark cycle and access to food and water ad libitum and maintained under pathogen-free conditions. The animal study was approved by the Institutional Animal Care and Use Committee (IACUC) at the Mayo Clinic.

Wild animals

Wild animals were not used in this study.

Field-collected samples

Field-collected samples are not part of this study.

Ethics oversight

The animal study was approved by the Institutional Animal Care and Use Committee (IACUC) at the Mayo Clinic (see Methods section).

Note that full information on the approval of the study protocol must also be provided in the manuscript.

Human research participants

Policy information about studies involving human research participants

Population characteristics	Archive tumor tissue collected at Mayo Clinic (Rochester, MN) and the Prostate Centre at the University of British Columbia (Vancouver, BC, Canada). Cases were selected solely based on their histopathology diagnosis and tumor tissue availability.
Recruitment	Samples were collected retrospectively from the Mayo Clinic (Rochester, MN) and the Prostate Centre at the University of British Columbia (Vancouver, BC, Canada) and annotated for major clinicopathologic variables through review of pathology reports and clinical records by trained personnel.
Ethics oversight	The studies were approved by the Institute Review Board (IRB) of the Mayo Clinic.

Note that full information on the approval of the study protocol must also be provided in the manuscript.

Dual use research of concern

Policy information about dual use research of concern

Hazards

Could the accidental, deliberate or reckless misuse of agents or technologies generated in the work, or the application of information presented in the manuscript, pose a threat to:

No	Yes	
☒	☐	Public health
☒	☐	National security
☒	☐	Crops and/or livestock
☒	☐	Ecosystems
☒	☐	Any other significant area

Experiments of concern

Does the work involve any of these experiments of concern:

No	Yes	
☒	☐	Demonstrate how to render a vaccine ineffective
☒	☐	Confer resistance to therapeutically useful antibiotics or antiviral agents
☒	☐	Enhance the virulence of a pathogen or render a nonpathogen virulent
☒	☐	Increase transmissibility of a pathogen
☒	☐	Alter the host range of a pathogen
☒	☐	Enable evasion of diagnostic/detection modalities
☒	☐	Enable the weaponization of a biological agent or toxin
☒	☐	Any other potentially harmful combination of experiments and agents

ChIP-seq

Data deposition

☒ Confirm that both raw and final processed data have been deposited in a public database such as GEO.

☒ Confirm that you have deposited or provided access to graph files (e.g. BED files) for the called peaks.

Data access links<br>*May remain private before publication.*	The ChIP-seq data are deposited in the National Center for Biotechnology Information (NCBI) Gene Expression Omnibus (GEO) database with the accession code GSE136130: https://www.ncbi.nlm.nih.gov/geo/query/acc.cgi?acc=GSE136130.
Files in database submission	The BigWig files of ChIP-seq data were submitted.
Genome browser session<br>(e.g. UCSC)	N/A

Methodology

Replicates	At least duplicates for all ChIP-seq data. AR, FOXA1 and H3K27ac ChIP-seq data were triplicated. CXXC5 and TET2 ChIP-seq data were duplicated.
Sequencing depth	Generally >30M reads for each ChIP-seq sample, around ~90% overall alignment rate, and 70-80% unique mapping ratio were

achieved. All these ChIP-seq samples were sequenced by paired-end with 51 bp in length.

Antibodies	ChIP antibodies were AR (2 μg/sample; #sc-816, Santa Cruz Biotechnology), FOXA1 (2 μg/sample; #ab23738, Abcam), H3K27ac (2 μg/sample; #ab4729, Abcam), CXXC5 (2 μg/sample; #16513-1-AP, Proteintech), TET2 (2 μg/sample; #ab94580, Abcam) and Rabbit IgG (2 μg/sample; #ab171870, Abcam).
Peak calling parameters	Peaks were called with MACS2 using default parameters with a cut off of P <1 e-5.
Data quality	ChIP-seq data quality was checked using FastQC. Poor quality reads and adapters were trimmed before further processing. Peaks that were called within a minimum of two or more biological replicates were retained to generate a consensus peakset and downstream analyses. We identified the total AR binding sites around 59,780 peaks, which is consistent with other reports in literature.
Software	1) Bowtie2 v2.1.0 with default parameters was used for reads mapping to GRCh37 (hg19). (https://sourceforge.net/projects/bowtie-bio/files/bowtie2/2.1.0/).<br>2) Callpeak function in MACS2 v2.0.10 was used peaks calling. (https://pypi.org/project/MACS2/2.0.10.20131216/).<br>3) AnnotatePeaks.pl in HOMER v4.11 was used for peaks' genomic distribution annotation with regard to TSS and target genes (http://homer.ucsd.edu/homer/ngs/annotation.html).<br>4) STEME v1.9.6 was used for de novo motif discovery (https://pypi.org/project/STEME/#files).<br>5) Tools of computeMatrix and plotHeatmap in deepTools 2.0 were used for heatmap (https://deeptools.readthedocs.io/en/develop/).
